# Household tenure and its associations with multiple long-term conditions amongst working age adults: a cross-sectional analysis using primary care and local government data linked at individual and household levels.

**DOI:** 10.23889/ijpds.v7i3.1776

**Published:** 2022-08-25

**Authors:** Elizabeth Ingram, Manuel Gomes, Sue Hogarth, Helen McDonald, David Osborn, Jessica Sheringham

**Affiliations:** 1 University College London (UCL); 2 London Boroughs of Camden and Islington; 3 London School of Hygiene and Tropical Medicine (LSHTM); 4 University College London (UCL)

## Objectives

We quantified associations between household tenure – whether someone privately or socially rents their property, or owner occupies – and prevalence of multiple long-term conditions (MLTCs) amongst working age adults. We also assessed whether the success of data linkages conducted to enable this study introduced potential selection biases.

## Approach

This cross-sectional study used the 2019/20 wave of an innovative dataset linking health and local government data at individual and household levels for residents of an East London borough. To assess potential biases, we calculated standardised differences in variables for matched and unmatched primary care records. Our primary outcome was basic MLTCs, defined as two or more long-term conditions from a list of 38. Two further definitions of MLTCs were operationalised. Multilevel logistic regression was used to explore associations for working age adults (16-64 years, inclusive). Interaction terms were used to evaluate potential interactions between tenure and other household factors.

## Results

Standardised differences in selected health variables for matched and unmatched primary care records were <0.2, indicating selection biases were not introduced due to data linkage success. For participants with successfully linked records, prevalence of basic MLTCs was 18.0%. After adjusting for various sociodemographic, health and socioeconomic variables, odds of basic MLTCs were 36% higher for working age social housing tenants (OR 1.36; 95% CI 1.30-1.42) and 19% lower for private renters (OR 0.81, 95% CI 0.77-0.84) when compared to owner-occupiers. These results were largely consistent across different definitions of MLTCs. Other household-level variables - household benefits receipt, occupancy, and household type – were important modifying factors, with associations between tenure and MLTCs greater for individuals in single adult households and households in receipt of benefits.

## Conclusion

This study demonstrates that primary care and local government data can be linked without introducing selection biases in key health variables and analysed to reveal important insights. We found evidence that household tenure is associated with MLTCs prevalence, emphasising the importance of understanding and addressing household-level social determinants of health.

